# Progress towards the Replacement of the Rabbit Blood Sugar Test for the Quantitative Determination of the Biological Activity of Insulins (USP <121>) with an In Vitro Assay

**DOI:** 10.3390/ani13182953

**Published:** 2023-09-18

**Authors:** Sabrina Rüggeberg, Antje Wanglin, Özlem Demirel, Rüdiger Hack, Birgit Niederhaus, Bernd Bidlingmaier, Matthias Blumrich, Dirk Usener

**Affiliations:** 1 CMC-Bioanalytics, R&D Sanofi, 65926 Frankfurt, Germany; 2 TIM Global Compliance and Policy, R&D Sanofi, 65926 Frankfurt, Germany; 3 CMC-Statistics, R&D Sanofi, 65926 Frankfurt, Germany; birgit.niederhaus@sanofi.com; 4 MSAT Analytics , M&S Sanofi, 65926 Frankfurt, Germany; 5 MSAT Project Nitrosamines, M&S Sanofi, 65926 Frankfurt, Germany

**Keywords:** USP <121>, insulin cell-based bioassay, in-cell western, ICW, rabbit blood sugar test, 3R, replacement, reduction, refinement, method bridging

## Abstract

**Simple Summary:**

The recent United States Pharmacopeia general chapter <121> requires a non-quantitative bioidentity test either as a rabbit blood sugar assay or as an in vitro insulin cell-based assay using in-cell Western (ICW) technology for insulin batch release. However, for quantification during stability or comparability studies, the rabbit blood sugar test is still required using a minimum of 24 rabbits to obtain one result. Based on the 3R principle (replace, reduce, and refine), this study sought to qualify the in vitro ICW cell-based bioassay approach for quantifying insulin activity. A bridging study with different insulins and stress samples revealed a clear correlation between the in vitro and in vivo test results. The replacement of the animal-based assay with the quantitative in vitro ICW cell-based bioassay for batch quality control saved cost, reduced cycle times while obtaining more meaningful and reliable data, and, above all else, reduced the suffering of many rabbits.

**Abstract:**

For the quantification of insulin activity, United States Pharmacopeia (USP) general chapter <121> continues to require the rabbit blood sugar test. For new insulin or insulin analogue compounds, those quantitative data are expected for stability or comparability studies. At Sanofi, many rabbits were used to fulfil the authority’s requirements to obtain quantitative insulin bioactivity data until the in vivo test was replaced. In order to demonstrate comparability between the in vivo and in vitro test systems, this study was designed to demonstrate equivalency. The measurement of insulin lispro and insulin glargine drug substance and drug product batches, including stress samples (diluted or after temperature stress of 30 min at 80 °C), revealed a clear correlation between the in vitro and in vivo test results. The recovery of quantitative in vitro in-cell Western (ICW) results compared to the in vivo test results was within the predefined acceptance limits of 80% to 125%. Thus, the in vitro ICW cell-based bioassay leads to results that are equivalent to the rabbit blood sugar test per USP <121>, and it is highly suitable for insulin activity quantification. For future development compounds, the in vitro in-cell Western cell-based assay can replace the rabbit blood sugar test required by USP <121>.

## 1. Introduction

The early concept of replacing, reducing, or refining animal use in research and testing was set in the late 1950s by Russell and Burch with their book *The Principles of Humane Experimental Technique* [1]. The concept of replacement aims to substitute traditional animal models with non-animal systems such as biochemical or cell-based systems, the concept of reduction aims to decrease the number of animals required for testing, and the concept of refinement attempts to eliminate pain or distress in animals and enhance animal well-being.

This 3R principle comprises the ethical guidelines for animal experiments, which are only allowed if no alternative method is available, as regulated by European legislation. The EU directive 2010/63/EU on the protection of animals used for scientific purpose is to be considered, and it represents “…*an important step towards achieving the final goal of full replacement of procedures on live animals for scientific and educational purposes as soon as it is scientifically possible to do so…*”.

Thus, the three Rs for animal-based experiments are a common goal of health authorities and pharmaceutical industries worldwide [2,3,4,5,6]. The European Union article 13 of the new directive 2010/63/EU further states that “*Member States shall ensure that a procedure is not carried out if another method or testing strategy for obtaining the result sought, not entailing the use of a live animal, is recognized under the legislation of the Union*” [7]. Consequently, the European Medicines Agency has recommended to marketing authorization holders to ensure compliance with the 3R methods described in the European Pharmacopoeia, and it points to the fact that competent authorities for granting the approval of animal testing will request the more animal-friendly European Pharmacopoeia method to be used [8].

Progress has been achieved for refinement [9,10], reduction [11], and replacement in research and as quality controls for vaccines [12] or pharma products [13].

Nevertheless, animal tests still play an essential role in the research (e.g., to assess toxicity [14]), development [15], and quality control of vaccines [16] or pharma products [17]. There are still legislative demands which require animal testing for quality control [10,15].

The animal-based biological assays such as the rabbit blood sugar test have a long history in the testing and development of human insulin and insulin analogues for release [18]. The current USP general chapter <121> requires the use of either the in vivo rabbit blood sugar test or the in vitro in-cell Western cellular assay with a specification of not less than 15 units (U) per mg of insulin as a qualitative bioidentity measurement for the release of insulin or insulin analogue batches manufactured for the United States market [19]. Quantitative measurements are needed to assess the long-term activity of insulin or an insulin analogue during stability or comparability studies. For these quantitative measurements, the in vivo rabbit blood sugar test is still mandatory. Alternatively, the in vitro in-cell Western (ICW) cell-based bioassay could be used for quantitative measurements by applying the same statistical significance as defined by USP general chapter <121>, with a confidence limit of ±10%.

Within Sanofi, the in vivo rabbit blood sugar test had still been performed for the development and approval of new insulins or insulin analogues to fulfil the regulatory requirements of authorities in several countries, such as the United States, China, and Japan. Until 2018, many thousands of rabbits were used to support these studies. Since 2018, Sanofi has relied on in vitro ICW cell-based bioassay data.

The in vitro ICW cell-based bioassay procedure was introduced in USP general chapter <121> in 2020 [19]. Its use and the ICW methodology have been described previously [13,20,21] in articles, and they were further characterized by the FDA [22] and alternative cell-based assays have been proposed [23]. The ICW method measures the biological effect of human insulin through the activation of the human insulin receptor (hIR). The binding of human insulin or insulin analogs to the hIR induces a conformational change that stimulates the auto-phosphorylation of the hIR on three tyrosine (Tyr) residues [24]. This auto-phosphorylation leads to the full activation of the hIR and enhances its activity towards the intracellular substrates involved in the downstream signaling cascade, leading to the metabolic end effects and, finally, to a decrease in blood sugar level [25,26]. Measuring the auto-phosphorylation of hIR reflects the activity of the hormone, and it can be used for mimicking the biological activity of human insulin or insulin analogs [24]. The initial step of human insulin action, the activation of hIR auto-phosphorylation, is a highly efficient read-out for such biological activity because it is a direct approach that leads to accurate data with low background noise.

The objective of this study was to demonstrate analytical equivalence between the in vivo quantitative rabbit blood sugar test according to USP <121> (in short, the in vivo test) and the in vitro quantitative bioassay by ICW (in short, the in vitro qICW).

The study was divided into two parts. The first part evaluated regular test items. The second part evaluated decreased potent/stressed test items with the in vitro qICW and the in vivo test.

## 2. Materials and Methods

### 2.1. Samples Set

#### 2.1.1. The First Part: The Regular Test Items

Samples from the primary stability studies of Suliqua (a lixisenatide/insulin glargine combination), the insulin lispro Sanofi drug product (DP), and the insulin lispro Sanofi drug substance (DS) were analyzed with the in vitro insulin cell-based bioassay and in vivo rabbit blood sugar test.

#### 2.1.2. The Second Part: The Stressed Test Items

The insulin lispro Sanofi DP (LISDP001) was stressed at 80 °C for 30 min or diluted to 50% of its initial concentration. The in vitro qICW and in vivo tests were performed to compare the loss of potency.

The relevant parameters (appearance of solution (i.e., clarity and color), assay insulin lispro by HPLC, product-related substances by HPLC, product-related impurities, and particulate matter (i.e., visible particles)) of the temperature-stressed insulin lispro were monitored to confirm the stability of the stress-induced changes.

### 2.2. Statistical Considerations

The objective of the evaluation was to demonstrate that the in vitro test results are statistically comparable to the in vivo test results. To achieve this, an equivalence approach was used as an appropriate statistical test (for the use of equivalence instead of difference testing, also see USP <1033> [27] and ICH-Q2 [28]) as set out below.

In order to compare the in vitro and in vivo test results, recoveries were calculated. The recoveries were calculated by dividing the in vitro qICW results by the in vivo test results (ratio), followed by a multiplication by 100 in order to obtain the percentage values, as follows:

$${\mathrm{recovery}}_{}=\frac{\mathrm{in}\;\mathrm{vitro}\;\mathrm{qICW}\;\mathrm{result}}{\begin{array}{c} \text{in vivo test result} \end{array}}\times 100.$$

A recovery of 100% therefore corresponded to identical results for the in vitro and in vivo tests. To statistically demonstrate equivalence between the two methods (in vitro and in vivo), a predefined acceptance criterion had to be applied to the mean recovery, with a 90% confidence interval, by applying the following decision procedure:

- the in vitro and in vivo test results were considered comparable at the 5% significance level and the in vitro tests were considered as performing acceptably if the 90% two-sided CI was totally within the acceptance interval
- the difference between the in vitro and in vivo test results was considered as significant in cases where the point estimates of the recoveries were outside the acceptance interval, and the performance of the in vitro test was considered as unsatisfactory for using the method
- there was insufficient information to conclude that there were no relevant differences between the in vitro and in vivo tests, beyond a reasonable doubt, in cases where the point estimates of the recoveries lied within the acceptance interval and the CI overlapped the acceptance interval, and the decision had to be based on the magnitude of the observed recovery and other supporting information (i.e., further experiments may have been needed)

The below acceptance criteria (see Table 1) needed to be met to demonstrate equivalency between the in vitro qICW and the in vivo test based on USP <1090> for demonstrating bioequivalency.

The number of measurements (i.e., the sample size) for showing equivalence was calculated to obtain accurate results with a power of 80%. A minimum of 12 measurements were needed for a valid result. In this study, 19 samples were tested with each method to demonstrate equivalency between the in vitro qICW and the in vivo test.

The mean recovery was calculated using the internally developed and validated software BioSt@t-Stars version 2.6. The in vitro qICW data were obtained on a logarithmic scale and the in vivo test data were obtained on a linear scale. Since the acceptance criterion was given on the logarithmic scale, further calculations were continued on the logarithmic scale.

### 2.3. In Vitro Cell-Based Bioassay using the In-Cell Western Cell-Based Method (USP <121> Method)

Chinese hamster ovary (CHO) cells expressing human insulin receptor B (hIR, genebank accession number M10051) (ATCC, Manassas, VA, USA, CRL-3307™) were cultivated with 90% Ham’s F12 nutrient mixture with glutamax, 10% fetal bovine serum, and 0.6% hygromycin B (Life Technologies, Darmstadt, Germany) at 37 °C in a humidified atmosphere of 5% CO_2_.

In order to determine the tyrosin (Tyr) phosphorylation status of the hIR [29], the cells were seeded into 96-well microplates with a density of ~0.5 to 1.5 × 10^5^ cells/mL and grown for 2 to 4 days. The cells were serum-starved with serum-free Ham’s F12 nutrient mixture with glutamax for 3 to 5 h at 37 °C in a humidified atmosphere of 5% CO_2_. The cells were subsequently treated with serial dilutions of human insulin or insulin analog prepared in serum-free Ham’s F12 nutrient mixture with glutamax (insulin glargine) or in 0.1% BSA in D-PBS (insulin lispro) for 20 min at 37 °C and 5% CO_2_. For the potency determination of the insulin or the insulin analog, two independent measurements of triplicates on different microplates were performed. An insulin reference standard was analyzed in parallel on each plate.

After stimulation, the medium was discarded and the cells were fixed in 3.7% freshly prepared para-formaldehyde (Merck, Darmstadt, Germany) in Dulbecco’s phosphate-buffered saline without calcium and magnesium (D-PBS; Life Technologies, Darmstadt, Germany) for 20 min. After permeabilization with 0.1% Triton^®^-X-100 (Merck, Darmstadt, Germany) in D-PBS for 2 × 10 min, blocking was performed with a blocking solution containing 2% bovine serum albumin (BSA; Sigma Aldrich, Taufkirchen, Germany) in D-PBS overnight at + 2–8 °C. Immersion in an incubation mixture with the anti-p-Tyr 4G10 mouse monoclonal antibody (Millipore, Schwalbach, Germany) [30] prepared in D-PBS and 0.1% (*v*/*v*) polysorbate 20 (AppliChem, Darmstadt, Germany) for insulin glargine or in 2% BSA in D-PBS and 0.1% (*v*/*v*) polysorbate 20 for insulin lispro for 2 h at room temperature was followed by a washing step with D-PBS containing 0.1% polysorbate 20. Incubation with an IRdye 800 CW goat anti-mouse IgG antibody (Li-Cor, Bad Homburg, Germany) and cell/DNA staining dye prepared in D-PBS and 0.2% (*v*/*v*) polysorbate 20 for insulin glargine or in 2% BSA in D-PBS and 0.2% (*v*/*v*) polysorbate 20 for insulin lispro was performed for 1 h at room temperature. The application of near-infrared-labeled antibodies had the distinct advantage of a high signal-to-noise ratio due to very little auto-fluorescence from both cellular materials and plastics. Fluorescence was detected by the Odyssey Infrared Imaging System (Li-Cor, Bad Homburg, Germany) using the 800 nm channel for the detection of the tyrosine phosphorylation at the hIR. The results were normalized to the cell number by combined cell- and DNA-staining with Sapphire700™ (Li-Cor, Bad Homburg, Germany) and DraQ5™ (BioStatus, Leicestershire, UK) and detected with the 700 nm channel.

The normalized data of the dilution curves of a reference standard and the test samples were used to perform a 4-parameter logistic (4-PL) regression analysis and calculate the EC_50_ value (the half maximal effective concentration), which represented the potency of the insulin and insulin analog samples. The relative potencies were calculated based on dividing the EC_50_ value of the reference standard by the EC_50_ value of the sample, which was then multiplied by 100%. The obtained relative potency was further calculated to units/mg (or U/mg) with the known activity of the reference standard. The final potency results were reported either as relative potencies in percentages or in U/mg.

The Suliqua and insulin lispro DS and DP were measured with the in vitro qICW method. For the Suliqua, eight replicate measurements for the in vitro qICW and insulin lispro were calculated using the same requirements, which are described in USP <121> for the in vivo test, with 95% CI ≤ 0.082 (CL within ±10%).

### 2.4. HPLC Method for Assay Quantification

The assays for the insulin, product-related substances, and product-related impurities, including the degradation products of the insulin lispro in the drug product, were quantified using validated reversed phase liquid chromatography (HPLC) methods at Sanofi (Frankfurt, Germany). The assay for the insulin was determined by external standard calibration whereas the product-related substances and product-related impurities, including the degradation products of the insulin lispro, were quantified by the 100% peak area method (i.e., the normalization method). Three independent determinations per testing time-point were performed for each sample, and the mean values of those measurements were calculated. As per internal Sanofi rules for analytics procedures, the individual measurements did not differ from each other by more than 2%.

### 2.5. HPSEC for Determination of High-Molecular-Weight Proteins

The determination of any impurity with a molecular mass greater than that of insulin was performed by size-exclusion chromatography as prescribed in the Ph. Eur. and USP. The use of the methods for the individual insulins was verified or validated, respectively. The quantification was performed by the 100% peak area method (i.e., the normalization method). The sum of the areas of the peaks with retention times less than those of the principal peaks (i.e., the peaks due to the insulin monomer) was regarded to be the sum of the high-molecular-weight proteins. Any peak with a retention time greater than that of the peak due to the insulin monomer was disregarded.

### 2.6. In Vivo Rabbit Blood Sugar Test

The experimental design and management procedures were approved by the District Government of South-Hesse in Darmstadt under animal use permit nos. HMR-7/Anz. 02 and FH-1009 according to the German Animal Welfare Legislation implementing the European Directive 2010/63/EU. The studies were conducted in an AAALAC International-accredited facility of Sanofi in Frankfurt.

#### 2.6.1. Animals and Husbandry

New Zealand White (NZW) female rabbits aged 10–16 weeks with body weights of at least 1.8 kg were purchased from two rabbit breeders (Bauer, Neuenstein, Germany and Zimmermann, Untergröningen, Germany). After arrival, the animals were kept in groups of a maximum of 32 animals in solid-floor pens with elevated platforms for at least 7 days before the study start and between the study parts. The animals were housed in environmental conditions as follows: a temperature range of 15–21 °C, a relative humidity rate of 40–70%, and an air change rate 18–21 times/hour. Water and food (sniff rabbit maintenance diet, Soest, Germany) were provided ad libitum, and hay and aspen sticks were given as environmental enrichment. All the animals were inspected daily by skilled personnel.

#### 2.6.2. Experimental Design

The quantitative rabbit blood sugar test was performed according to USP general chapter <121>. For the testing of one test article, the rabbits were randomly assigned to 4 groups of at least 6 animals.

Fourteen hours before the dosing food was withdrawn and approximately 1 h before dosing, the animals were transferred into a rabbit restrainer and arterial catheters were implanted in their central ear arteries. Two solutions of standard preparations and two solutions of test articles were prepared and applied in volumes of 0.5 mL subcutaneously to two respective groups of rabbits.

At 1 and 2.5 h, blood samples (1.3 mL) were collected from the rabbits’ central ear arteries into tubes coated with fluoride-heparin (microtubes from Sarstedt). After blood sampling, the catheters were removed, and the animals were brought back to their home areas. The whole period in the restrainer normally lasted 3.5 h.

**Figure 1:**
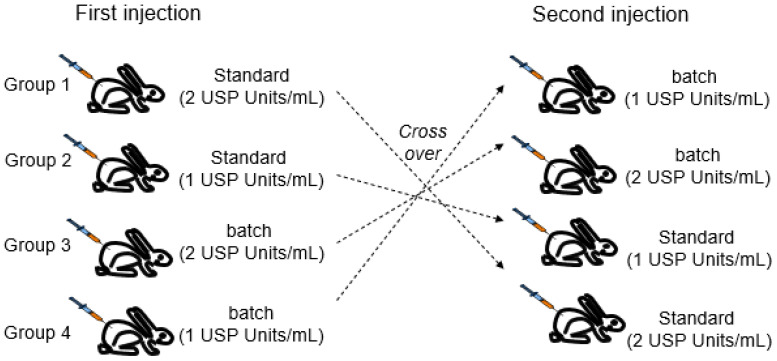
Double cross-over design of the rabbit blood sugar test according to USP <121>.

Two to six days later, the second part of the study was performed using a twin cross-over design (see Figure 1).

The human insulin and the other insulin analogues used are xenoproteins for rabbits, and as with all xenoproteins, there is a risk of the production of anti-drug antibodies (ADA) against these xenoproteins, especially after several exposures. These ADAs were considered as a risk for under-estimating the activity of the insulin in the samples, and therefore, they represent a risk for patients who receive too much insulin. Finally, after extensive discussions with immunologists and the animal welfare authority, it was decided to re-use the animals once. After the second test, the possibility of animal re-use for the other rabbit experiments was evaluated. If re-use was not possible, then the animals were handed over to a local zoo in accordance with local veterinarian authorities to be used for the feeding of carnivores.

After blood centrifugation, the plasma samples were analyzed for their blood glucose concentrations with the Hexokinase method using a multianalyser (KonelabPrime 30) (Thermo Fisher, Dreiech, Germany). The basic principle of this method is the phosphorylation of glucose to glucose-6-phosphate in the presence of ATP and hexokinase, followed by the oxidation of glucose-6-phosphate to 6-phosphogluconate by glucose-6-phosphatedehydrogenase. In this reaction, an equimolar amount of NADP was reduced to NADPH2, with a resulting increase in the absorbance at 340 nm. The increase was measured by the KonelabPrime 30 instrument.

The final calculations were completed according to USP <121>. The 95% confidence interval of the final result needed to be smaller than 0.082, which corresponded to confidence limits of approximately ± 10%. For all regular test items, the in vivo test was performed in the course of the respective stability studies.

## 3. Results

The regular test items and stressed test items were tested, and the results are summarized below.

### 3.1. Regular Test Items

Nineteen different insulin/insulin analogue batches were tested during the stability studies in order to cover the different conditions such as the storage temperature (−20 °C, 5 °C, 25 °C, 37 °C, and 40 °C), age (freshly produced (T0), 36 months), formulations (DS and DP), and concentrations (approximately 25 U/mL to 100 U/mL). Two different insulin analogues (insulin lispro and insulin glargine) were used for this study. All the single results for the contents of the bioactive insulins are listed in Table 2. The individual recoveries varied between 80.11% to 118.75%. The mean recovery of the regular test items was 95%, with a 90% confidence interval (CI) ranging from 91% to 99% and a geometric coefficient of variation (CV%) of 12% (95% one-sided upper confidence limit (CL) of 17%).

**Figure 2:**
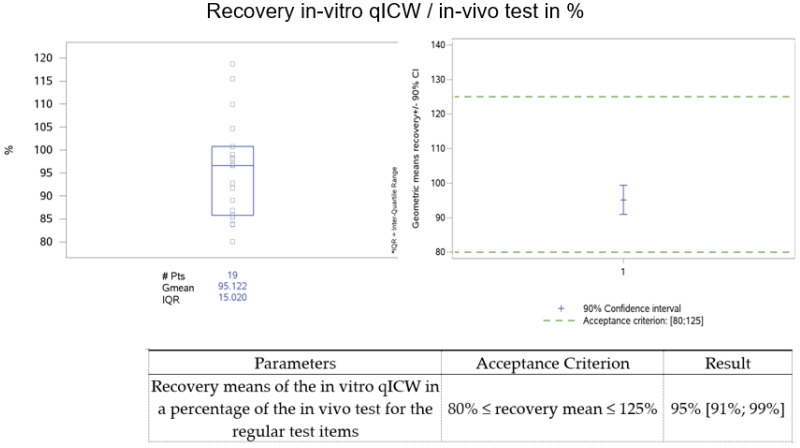
The results for the mean recovery and the 90% CIs for the regular test items are displayed. A total number of 19 drug substance and drug product batches of either insulin lispro or insulin glargine were compared by calculating the recovery of the in vitro qICW concentration (U/mg) in the in vivo test concentration (U/mg), as shown in Table 2.

The data were statistically analyzed, as shown in Figure 2. The 19 final recovery results with the geometric mean in the interquartile range are given in a box blot for the data.

### 3.2. Stressed Test Items

Different reduced biological potencies were determined using either the stressed or the diluted samples. As for the regular test items, the recoveries were calculated to show similarity. Furthermore, a set of physicochemical tests were performed to demonstrate the sample suitability for the study and to monitor the heat-stressed test items.

As these measurements were dedicated only to this study, the number of experiments was kept as low as possible to comply with the 3R principle for the in vivo tests.

The results of the physicochemical characterization are listed in Table 3, and they show highly stressed insulin lispro. The amount of the product-related substance 3^B^-Asp insulin increased to 4.28% from 0.79%, and other impurities increased to 1.78% from 0.35% while the aggregates (i.e., the high-molecular-weight proteins) increased to 32.48%.

All the single results obtained for the contents by HPLC and the bioactivity by the in vitro qICW or the in vivo test are listed in Table 4. While the unstressed starting material revealed potencies of 101.28 U/mL and 103.3 U/mL as determined by the in vitro qICW and the in vivo rabbit blood sugar test, respectively, the recovery was calculated to be 98.04%. Lower potency values, either by dilution (47.45 vs. 51.87 U/mL) or stress (63.59 vs. 64.77 U/mL), showed very good recovery rates of 91.48% and 98.18%, respectively.

**Figure 3:**
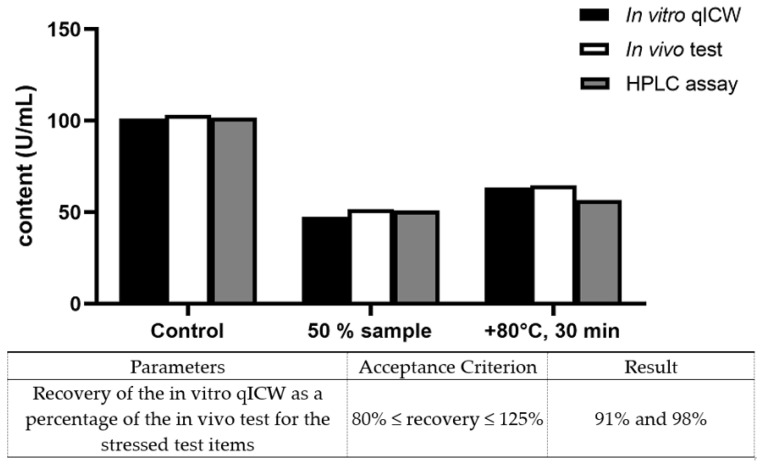
The results for the stressed and unstressed insulin lispro Sanofi LISDP001 and the recoveries.

The clear correlation between the in vitro qICW and the in vivo rabbit blood is visualized in Figure 3. The contents in U/mL are given for the unstressed items on the right, for 50% potency in the middle, and for the stressed insulin lispro Sanofi on the left. The in vitro qICW test system is shown in black, the in vivo rabbit blood sugar test is shown in white, and the RP-HPLC contents are shown in grey.

Thus, the stressed samples method confirmed the similarity between the in vitro qICW and the in vivo rabbit blood sugar test.

## 4. Discussion

All acceptance criteria were met for the regular test items, with a mean recovery of 95% and a 90% confidence interval of 91% to 99%. Furthermore, each single recovery met the predefined acceptance criteria. It could be concluded that the in vitro qICW and the in vivo test deliver similar results.

Seven different DP batches of either insulin glargine or insulin lispro and three different DS batches of insulin lispro were used for this study. The in vivo tests were performed on a regular basis for the primary stabilities during development. No additional in vivo tests could be performed for the regular test items due to animal welfare regulations and ethical reasons. Hence, the in vivo data from existing studies were used. The in vitro qICW data for Suliqua consisted of the initial Bio-ID (two plates) combined with additional experiments (six plates) to reach USP <121> precision, and all plates of the in vitro qICW for the insulin lispro Sanofi DS and DP were performed at the same time. The insulin lispro batch LISDP001 was tested with fewer replicates, which led to higher variability in the test results and represented a worst-case scenario for this bridging study.

Many of the in vitro qICW results showed lower potencies than those of the in vivo tests. It appeared that the in vitro qICW was more susceptible to stability-induced changes in the insulin lispro.

The acceptance criterion of a ≤20% difference (0.0792 on a log scale) was equivalent to the acceptance criterion of an 80% ≤ recovery ≤ 125%. For the purpose of harmonization, the acceptance criterion expressed as 80% ≤ recovery ≤ 125% is used.

Both stressed test items met the acceptance criterion of 80% ≤ recovery ≤ 125%, with recovery rates of 91% (the 50% test item) and 98% (the heat-stressed test items).

The 50% test item was used as a positive control for the potency reduction. Both the in vitro qICW and the in vivo test showed potency results very close to 50%.

The heat-stressed test items showed slightly increased potencies (from 7% to 8%) with the in vitro qICW and the in vivo tests compared to the HPLC data. This may have been caused by degraded or aggregated insulin lispro molecules which were still functional.

Potency measurements are needed to reflect the integrity of a complex three-dimensional structure in a solution, and this cannot be measured using chromatographic techniques [31]. Thus, for biotherapeutics, a potency assay for measuring biological activity is required for all release, stability, or study measurements [32]. As insulin/insulin analogues are manufactured with very well-established and robust processes and are characterized by well -defined and stable conformational structures, HPLC data could be representative of the biological activity. Therefore, many authorities rely on physicochemical determination for the biological activity of an insulin [18]. Nevertheless, insulins are a class of biotherapeutics where the integrity determines the biological activity, clinical efficacy, and safety.

The biological activities of human insulin and insulin analogues are currently assessed by the in vivo rabbit blood sugar test for quantitative determinations [19]. The in vitro cell-based ICW bioassay based on detecting human insulin receptor auto-phosphorylation in intact cells as the first step of the insulin signaling pathway is quite representative of the mode of action already used for batch release bioidentity measurements [13,19,21,22].

This ICW as a quantitative version (qICW) was investigated as an equivalent test system to the in vivo rabbit blood sugar bioassay test for human insulin and insulin analogues. At Sanofi, several thousand rabbits per year (ranging from 1000 to 4000) were used for quantitative insulin bioassay determinations depending on the number of studies performed in research and development until the rabbit blood sugar test was replaced with the proposed in vitro insulin cell-based bioassay (qICW). Thus, this alternative should also be applied by other companies developing or producing insulin for marketed use.

Furthermore, this ICW is more precise, at least as accurate, and as robust as the in vivo rabbit blood sugar bioidentity test, and it allows for quantitation of the results with high reproducibility, which is beneficial for patients.

The assay is favorable from an ethical point of view because it can replace animal testing for the quality control of new insulin batches. This is especially relevant since many initiatives are ongoing and great efforts are being made to replace, reduce, and refine animal-based testing in line with the 3R principle.

Nevertheless, this replacement does not replace all animal experiments as, for example, in vivo toxicity tests (e.g., embryotoxicity, reproductive toxicity, carcinogenicity, etc.) are still needed to assess the safety profiles of new insulin analogues, for example, those that have been performed for insulin glargine [33] and insulin lispro [34].

In summary, the in vitro qICW can be regarded as a superior alternative to the currently prescribed rabbit blood sugar test to quantify the biological activities of insulins and insulin analogues.

## 5. Conclusions

All batches tested to demonstrate method similarity were within the predefined acceptance criteria, as shown in Figure 2 and Figure 3. Thus, the in vitro test system is similar and comparable to the in vivo test system for testing insulin bioassay activity according to USP general chapter <121>.

The data in this study clearly indicated that the rabbit blood sugar test can be replaced with the in vitro insulin cell-based bioassay (ICW) for the quantitative measurement of new insulins or insulin analogues during development or after post-approval changes. This will reduce the suffering of many rabbits and provide more meaningful and precise data for the determination of insulin potency for patient safety and efficacy.

## Figures and Tables

**Table 1:** Acceptance criteria for showing similarity.

Parameters	Acceptance Criterion
Recovery means of in vitro qICW in a percentage of the in vivo tests for the regular test items	80% ≤ recovery mean ≤ 125%
Recovery of in vitro qICW in a percentage of the in vivo test for the stressed test items *	80% ≤ recovery mean ≤ 125%

*, described as ≤20% of the difference (0.0792 on a log scale).

**Table 2:** Single results of each measurement and recovery (the in vitro qICW and the in vivo test) for each insulin lispro and insulin glargine batch.

	Batch	Comment		Content–In Vitro qICW (U/mg)	Content–In Vivo Test (U/mg)	Recovery: In Vitro qICW/ In Vivo Test (%)
Insulin lispro Sanofi	LISDP001	5 °C, t_0_	DP	101.28 *	103.30	98.04
	LISDP001	37 °C, 1 month	DP	89.48 *	104.85	85.34
	LISDP001	25 °C, 6 months	DP	95.48 *	98.48	96.85
Insulin Suliqua (lixisenatide/insulin glargine combination)	GLADP001	5 °C, 12 months	DP	118.76	113.41	104.72
	GLADP002	5 °C, 12 months	DP	123.63	104.11	118.75
	GLADP003	5 °C, 12 months	DP	125.00	108.18	115.55
	GLADP004	5 °C, t_0_	DP	107.04	106.18	100.81
	GLADP005	5 °C, t_0_	DP	93.16	111.07	83.88
	GLADP006	5 °C, t_0_	DP	93.97	105.48	89.08
	GLADP005	40 °C, 1 month	DP	108.03	98.27	109.93
	GLADP004	5 °C, 12 months	DP	108.03	109.04	99.07
	GLADP005	5 °C, 12 months	DP	109.02	110.85	98.35
	GLADP006	5 °C, 12 months	DP	102.87	122.77	83.79
Insulin lispro Sanofi	LISDS001	−20 °C, 24 months	DS	27.4	28.4	96.64
	LISDS002	−20 °C, 24 months	DS	27.5	30.0	91.72
	LISDS003	−20 °C, 24 months	DS	24.9	29.0	85.79
	LISDS001	−20 °C, 36 months	DS	26.2	28.2	92.81
	LISDS002	−20 °C, 36 months	DS	26.1	30.0	86.88
	LISDS003	−20 °C, 36 months	DS	23.2	29.0	80.11

* the in vitro qICW data were obtained with four replicates based on USP <1032> requirements [27].

**Table 3:** HPLC results for the heat-stressed LISDP001.

Test	Acceptance Criterion *	Results from the Certificate of Analysis (CoA)		Results from the Heat-Stressed Test Items	
**Assay insulin lispro (HPLC)**	3.30 to 3.64 mg/mL (95.0% to 105.0% of the label claim)	3.53 mg/mL (101.7% of the label claim) 101.7 USP insulin lispro units/mL		1.97 mg/mL (56.7% of the label claim)	
**Product-related substances (HPLC)**					
Total of 3^B^-Asp insulin lispro and 3^B^-iso-Asp insulin lispro	≤1.5%	0.79%		4.28%	
27^B^-allo-Thr insulin lispro	≤0.5%	0.35%		0.24%	
**Product-related impurities and degradation products (HPLC)**					
21^A^-Asp insulin lispro	≤1.00%	0.21%		0.27%	
Any other unspecified unidentified impurity	≤0.5%	RRT **	%	RRT **	%
		0.90	0.07%		
		0.95	0.06%	1.25	1.78%
		1.10	0.07%		
		1.17	0.05%		
		1.15	0.10%		
Total of other impurities	≤2.00%	0.34%		7.40% ***	
**High molecular weights**	≤0.50%	0.10%		32.48% ***	

* only for scientific information; ** RRT (relative retention time); *** the observed level was found to be out of the validated range of the analytical method; Asp and Thr refer to aspartic acid and threonine, respectively, at amino acid positions 3, 21, or 27.

**Table 4:** Results and calculated ratios of the stressed test items.

Substance	Batch	Comment		Content–In Vitro qICW (U/mL)	Content– In Vivo Test (U/mL)	Recovery: In Vitro qICW/ In Vivo Test (%)	Content–HPLC Assay (U/mL)
Insulin lispro	LISDP001	No stress	DP	101.28 ^a^	103.30	98.04	101.7 ^b^
Insulin lispro	LISDP001	50% diluted sample	DP	47.45	51.87	91.48	50.9 ^c^
Insulin lispro	LISDP001	80 °C, 30 min (heat stress)	DP	63.59	64.77	98.18	56.7

^a^, data taken from the stability start; ^b^, data taken from the release certificate; ^c^, initial value of 101.7 U/mg divided by 2 to obtain the 50% content.

## Data Availability

Not applicable.
